# Untangling the relationship between hemoglobin, peak troponin level, and mortality in patients with myocardial infarction

**DOI:** 10.17305/bjbms.2021.6744

**Published:** 2022-02-18

**Authors:** Vojko Kanic, Gregor Kompara, David Suran, Nina Glavnik Poznic

**Affiliations:** Department of Cardiology and Angiology, Division of Internal Medicine, University Medical Center Maribor, Maribor, Slovenia

**Keywords:** Baseline hemoglobin, myocardial infarction, myocardial injury, infarct size, percutaneous coronary intervention, body surface area, outcome, mortality

## Abstract

Patients with a history of myocardial infarction (MI) and lower admission hemoglobin (aHb) levels have a worse outcome than patients with higher aHb, but lower or similar peaks in enzymatic infarct size. Hemoglobin levels are positively correlated with body surface area (BSA), which is positively correlated with cardiac mass. We hypothesized that patients with lower aHb suffer comparatively greater myocardial injury. We examined the relationships between aHb, and troponin (Tn) normalized to BSA (Tn/BSA) and its association with 30-day mortality. Data from 6055 patients, who were divided into seven groups based on their aHb at 10g/L intervals, were analyzed, and the groups were compared. The relationships between aHb and Tn/BSA and between Tn/BSA and 30-day mortality were assessed. Patients with higher aHb levels had greater BSA (*p*<0.0001). A negative relationship between aHb and log10Tn/BSA was observed in the entire group, and in men and women separately (*p*<0.0001, *p*<0.0001, and *p*=0.013, respectively). The log10Tn/BSA value was associated with 30-day mortality in the entire group, and in men and women separately (*p*<0.0001, *p*=0.014, and *p*<0.0001, respectively). Our finding suggests that a similar peak Tn value in patients with lower aHb means comparatively greater myocardial injury relative to cardiac mass. This hypothesis helps to explain the worse outcomes in patients with lower aHb. According to our findings, Tn should be indexed to BSA to provide comparable information on cardiac injury relative to cardiac mass. Whether this relationship is causal remains to be clarified.

## INTRODUCTION

Substantial evidence from previous studies suggests that low admission hemoglobin (aHb) levels in patients with myocardial infarction (MI) are a predictor of poor outcome [1-5]. However, the data on myocardial injuries in different aHb classes in these patients are contradictory and paradoxical. Some studies have found similar peaks in enzymatic infarct size in different aHb classes [5], while others found even lower peaks in enzymatic infarct size in patients with lower aHb levels, although these patients had significantly higher mortality rates [4,6]. At present, available data suggest that lower aHb levels are associated with a similar or lower enzymatic infarct size but higher mortality rates after MI than higher aHb levels [4-6]. This contradicts the fact that higher troponin (Tn) levels are associated with larger myocardial injuries and poorer outcomes [7,8]. The association between aHb levels and outcome after MI was interpreted as being independent of the severity of the myocardial injury and multifactorial [9,10], but this was not satisfactorily explained [4-6]. The peak Tn level provides an estimate of the extent of myocardial necrosis [8]. If two patients have similar peak Tn values but different cardiac mass, they would have different infarct sizes relative to cardiac mass, and these similar Tn values would be expected to have different clinical consequences in these patients.

It is known that the hemoglobin mass is positively associated with body surface area (BSA) in men and women [11,12]. The cardiac mass (weight) is also positively correlated with BSA, and BSA is used as a biometric unit for normalizing left ventricular mass [13-16]. Hence, patients with higher aHb levels would be expected to have a larger BSA, and a larger left ventricular mass [13-17]. The reason for indexing physiological parameters to BSA is that BSA has been shown to correlate more closely with physiological parameters than the body weight does [13,14].

We hypothesized that patients with lower aHb levels would have greater cardiac injury normalized to BSA (which correlates with cardiac mass), and thus a relatively larger infarct size relative to cardiac mass and a less favorable outcome than patients with higher aHb levels. We could not find any data regarding the possible association between aHb levels and cardiac Tn normalized to BSA (Tn/BSA) in patients with MI who had undergone percutaneous coronary intervention (PCI). Our aim was to investigate the possible association between aHb levels in selected patients with MI, treated with PCI, and Tn/BSA and the association between Tn/BSA and 30-day mortality.

## MATERIALS AND METHODS

A retrospective observational study was conducted at the University Medical Center Maribor, an academic medical center with a 24/7 PCI service. We screened 7344 consecutive patients with MI who underwent PCI between 2007 and the end of 2017. Patients with aHb <100 g/L (302) and aHb ≥170 g/L (87) and patients with missing data for Tn (154) or BSA (746) were excluded, leaving 6055 patients whose baseline and treatment data were obtained from patients’ electronic medical records. Patients with aHb <100 g/L were excluded because of possible concomitant diseases (malignancy, chronic diseases.) that could influence the outcome, and patients with aHb levels ≥170 g/L were excluded because only one woman was found in this category. Patients were stratified according to their aHb level into seven groups at 10 g/L intervals in the aHb range 100-169 g/L. These aHb groups were compared.

Tn levels were determined at admission and at least once in the first 24 hours. After that, serum Tn levels were measured at various times at the discretion of the treating physician. TnI was determined by the chemiluminescence immunoassay method on Siemens Dimensions Vista Systems (Siemens Healthcare Diagnostics, DE, USA) with the reference interval <0.045 μg/L. The highest serum Tn level during the entire hospitalization was defined as the peak Tn level. Patients were treated according to published guidelines for the management of MI [7,18]. Thrombolysis has not been used in our region since 2003, and no rescue PCI was performed. The PCI strategy and concomitant medication were at the discretion of the operator and the attending physician.

The exact data on deaths were provided by the Slovenian National Cause of Death Registry. The study was approved by the University Medical Center Maribor Committee for Medical Ethics (Reference: UKC-MB-KME-59/19) and it fulfills the requirements of the Declaration of Helsinki.

### Definitions

The MI in patients was confirmed by a history of chest pain, diagnostic electrocardiographic changes, and a serial elevation of Tn above the 99^th^ percentile of the upper reference limit in our laboratory according to published guidelines [7,18]. The DuBois and DuBois formula was used for calculation of BSA [13]. Renal dysfunction on admission was defined as an estimated glomerular filtration rate (GFR) ≤60 mL/kg/1.73m^2^. The Bleeding Academic Research Consortium (BARC) bleeding criteria and BARC 3a bleeding (Hb drop of 30-50 g/L or any transfusion) were used [19]. Unsuccessful PCI was defined as Thrombolysis in Myocardial Infarction (TIMI) flow grade 0/1 after PCI. Heart failure was defined according to clinical criteria (bilateral pulmonary rales, S3 gallop, and edema) and/or radiological evidence of interstitial or alveolar edema requiring diuretic therapy.

### End points

The end points were the possible association between the aHb level and the peak cardiac Tn level normalized to BSA (Tn/BSA) and association between the Tn/BSA and 30-day all-cause mortality. In addition, we assessed the BSA in different patient groups according to the aHb levels.

### Ethical statement

Ethical, governance, and waiver of consent approvals were granted by the University Medical Center Maribor Committee for Medical Ethics (Reference: UKC-MB-KME-59/19), and all methods were performed in accordance with relevant guidelines and regulations.

### Statistical analysis

Continuous variables were summarized using the arithmetic mean with standard deviation for normally distributed data or the median with interquartile range for non-normally distributed data. The normality of distribution was verified using the Kolmogorov-Smirnov test. Fischer’s exact chi-square test was used for the comparison of categorical data. Continuous variables following a normal distribution were compared using the independent samples t-test, while non-normally distributed variables were compared using the Mann-Whitney U test.

Baseline characteristics of patients across the different categories were examined by Chi-square tests for categorical variables and by the ANOVA test with Bonferroni post hoc analysis for continuous variables. Because of the highly skewed results for the Tn values, we transformed Tn results to log10, then normalized log10 Tn values to BSA (log10 Tn/BSA) and used this variable. Logistic regression models were used to determine the possible independent association between log10Tn/BSA and 30-day mortality. In addition to the peak log10Tn/BSA value, variables with significant univariable association (*p*<0.05) with 30-day mortality and variance inflation factor <1.6 were included in the multivariable model. Models were adjusted for: age, sex, diabetes, hypertension, hyperlipidemia, renal dysfunction on admission, ST-elevation MI, aHb level groups, radial access, PCI of the left main coronary artery, left anterior descending artery, circumflex artery, right coronary artery, TIMI flow grade 0/1 after PCI, heart failure, dual antiplatelet therapy, bleeding, and log10Tn/BSA. In addition, we analyzed these possible associations for each sex separately. Data were analyzed with SPSS 25.0 software for Windows (IBM Corp, Armonk, NY).

## RESULTS

### Descriptive data for patients

A total of 6055 patients (69.9% men) with MI were included in the study. Divided into groups based on aHb levels, there were 315 (5.2%), 665 (11.0%), 1074 (17.7%), 1426 (23.6%), 1371 (22.6%), 879 (14.5%), and 325 (5.4%) patients in the aHb groups 100-109 g/L, 110-119 g/L, 120-129 g/L, 130-139 g/L, 140-149 g/L, 150-159 g/L, and 160-169 g/L, respectively (Table 1).

**TABLE 1:**
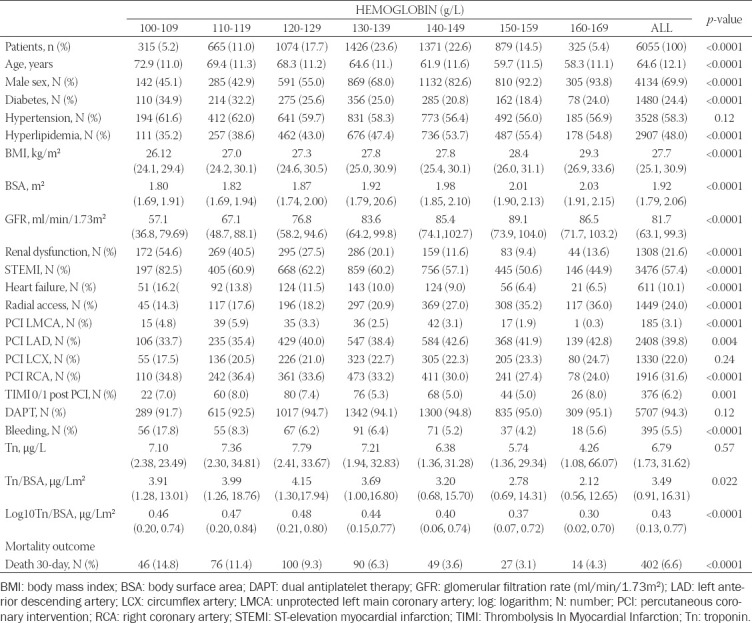
Patients with myocardial infarction stratified by baseline hemoglobin

Table 1 shows the clinical and procedural characteristics of the patients stratified by aHb levels. Patients with lower aHb levels were more likely to be female, older, and with ST-elevation MI on admission. They had a lower GFR and more frequently suffered from diabetes, but hyperlipidemia was less common. PCI of the left main coronary artery was performed more frequently, whereas PCI of the LAD was less often carried out. Radial access was less frequently used, and they were more prone to bleed. The BMI and BSA were generally lower, while the Tn/BSA and log10Tn/BSA values were usually higher, and heart failure was more frequent in patients with lower aHb levels.

### Graded hemoglobin level on admission and outcomes

#### BSA

Spearman’s correlation coefficient between the aHb level and BSA showed a positive correlation (r=0.358; *p*<0.0001). This correlation was stronger in men (r=0.214; *p*< 0.0001) than women (r=0.132; *p*<0.0001).

**FIGURE 1:**
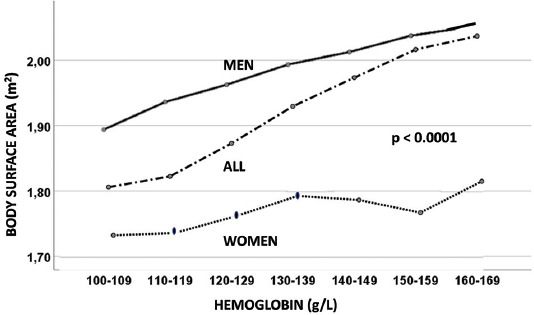
Relationship between hemoglobin level on admission and body surface area.

A positive relationship was found between aHb and BSA in the entire patient group (Table 1, Figure 1). A gradual increase in the aHb level was associated with a progressive increase in BSA (*p*<0.0001). This association was significant in both men and women when calculated separately (*p*<0.0001) (Figure 1).

#### Tn peaks

Peak Tn values were similar (p=0.57) in the different aHb groups (Table 1). When the log10Tn peak was normalized to the BSA value, the Spearman correlation coefficient between the aHb value and log10Tn/BSA showed a negative correlation (r=–0.107; p<0.0001). This negative correlation was stronger in men (r=–0.110; p<0.0001) than women (r=–0.087; p<0.0001).

**FIGURE 2:**
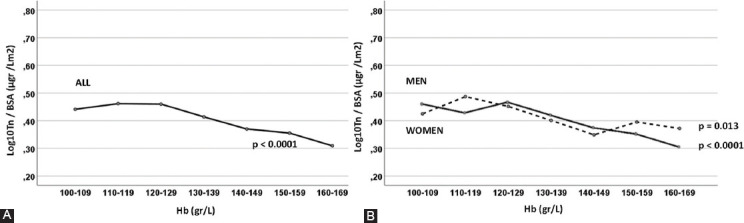
(A and B) Relationship between aHb level and peak log10Tn normalized to BSA (log10Tn/BSA). aHb: hemoglobin level on admission; Log: logarithm; Tn: troponin BSA: body surface area.

When we evaluated the univariable relationship between log10Tn/BSA and aHb in the entire group using ANOVA, similar results were observed in the aHb groups up to 120 g/L (Bonferroni pairwise comparison), and then a progressive decrease in the log10Tn/BSA value was associated with a progressive increase in the aHb level. Across the aHb range, this inverse relationship was significant (p<0.0001) (Figure 2A). This relationship was significant in both men and women (p<0.0001 and p=0.013, respectively) (Figure 2B).

#### Tn/BSA and mortality

A total of 402 (6.6%) patients died within 30 days (Table 1). Patients who died had Tn/BSA values of 10.15 μg/Lm^2 (^95% CI: 2.70-39.68) compared to 3.33 μg/Lm^2 (^0.85-15.12) in patients who survived 30 days (*p*<0.0001). Similarly, log10Tn/BSA values in patients who died (0.67 μg/Lm^2^ [0.38-0.99]) were significantly higher than those in patients who survived (0.41 μg/Lm^2^ [0.11-0.75]; *p*<0.0001). After multivariable adjustments, the log10Tn/BSA value was independently associated with 30-day mortality in the group of all patients and in men and women separately (adjusted OR 2.07; 95% CI: 1.51-2.85, *p*<0.0001; adjusted OR 1. 67; 95% CI: 1.11-2.53, *p*=0.014; adjusted OR 2.79; 95% CI: 1.66-4.68, *p*<0.0001, respectively) (Table 2). The Hosmer–Lemeshow test of goodness of fit indicated that the model was a good fit to the data (Chi-square=4.275, df=8, *p*=0.832).

**TABLE 2:**
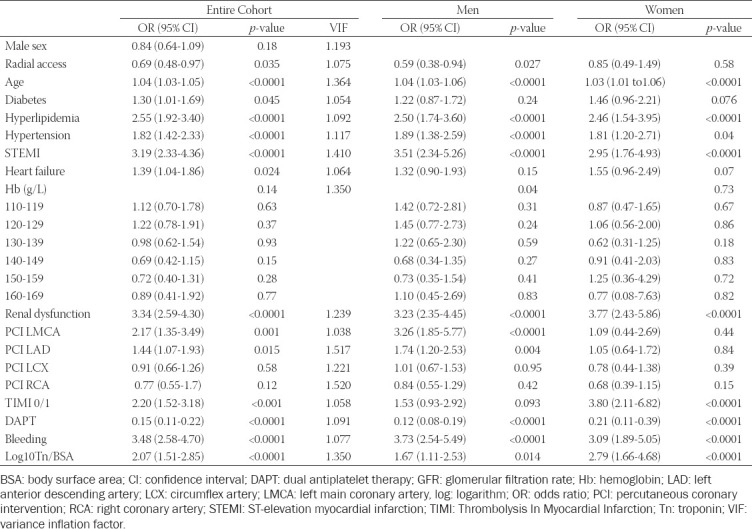
Predictors of 30-day mortality (group with aHb 100-109 g/L as a reference)

## DISCUSSION

Data on the relationship between aHb levels and cardiac injury in patients with MI are contradictory and paradoxical [4-6,9]. We investigated the association between aHb levels in patients with MI treated with PCI and their possible association with Tn/BSA, as well as the association between Tn/BSA and 30-day mortality.

The main results of our study are that the Tn/BSA was independently associated with 30-day mortality and that a gradual increase in aHb levels was associated with a decrease in the peak Tn/BSA.

In line with previous observations, we also observed a positive relationship between the aHb level and BSA [11,12], which is positively associated with cardiac mass [13-16]. A positive relationship was found between aHb and BSA in both sexes (Figure 1).

Our results show that in patients with MI, individuals with higher aHb levels had a higher BSA and a greater cardiac mass. Similar peak Tn levels, which correlate with necrotic myocardial mass, are likely to cause less adverse effects in larger compared to smaller hearts. Our finding suggests that Tn should be indexed to BSA (Tn/BSA) to provide comparable information on the extent of cardiac damage in relation to cardiac mass.

The positive relationship between aHb and BSA may not necessarily be causatively linked. Patients with lower aHb levels were older, predominantly women, and had a lower BMI. These are all well-known groups associated with a lower BSA, making this relationship very probable [13,20]. However, this relationship also has strong biological plausibility. The rate of red cell production is associated with the ability of the cell to deliver oxygen to the tissues relative to the tissue demand for oxygen [12]. A larger body size results in higher metabolic demands. Some data indicate that a higher erythrocyte count and a higher hemoglobin mass, together with a larger heart size and a greater left ventricular mass meet these demands [12,14,21].

Our finding that patients with lower aHb levels had higher peak log10Tn/BSA values was significant in both men and women (Figure 2B). This negative correlation was more pronounced in men. Possible mechanisms for this sex difference include differences in the pathophysiology of MI between the sexes, differences in presentation, unique sex-specific biology, and the possibility that the biological significance of the same aHb value differs between women and men [22-24]. The average age of women in our study was 7 years older than the average age of men, and a correlation between age and indexed left ventricular mass has previously been found only in women [16]. These possible differences need further investigation, which is beyond the scope of our analysis. Nevertheless, a higher log10Tn/BSA value was associated with higher mortality regardless of sex.

The higher risk of relatively larger infarct size in patients with lower aHb levels can be explained by several physiological mechanisms. Low aHb levels increase the supply-demand mismatch of oxygen in the myocardium and worsen myocardial ischemia due to the compensatory increase in stroke volume and a faster heart rate [25,26]. Low aHb levels also lead to a redistribution of blood flow from the subendocardial to the subepicardial myocardial layers, and together this can trigger myocardial necrosis [27,28]. In the setting of MI, this can promote arrhythmias, which further impair the already compromised myocardium [10,29]. Lower aHb levels not only decrease oxygenation of the injured myocardium, which is associated with a risk of increased infarct size but are also an integrator of many comorbidities and older age [30].

Relatively larger infarct size would be expected to lead to a higher mortality rate in patients with lower aHb, as determined in both our analysis and previous studies [4-6]. In line with these expectations, we observed a negative correlation between aHb and heart failure (r=–0.084; *p*<0.0001), as was previously observed in subgroups with lower aHb [4,9]. In contrast to the result of Archbold et al., we showed that heart failure and worse outcomes could be explained by the relatively larger infarct size [9]. Although most studies observed higher enzymatic peak infarct size in patients with higher aHb levels, the peak Tn level was similar in all aHb groups in our analysis [4,6]. Previous studies observed a myocardial band of creatine kinase that might explain the difference. However, comparisons must consider different patient selection, different patient aHb groups, and different treatment.

There is an ongoing debate as to whether a low aHb level is just a marker of the baseline clinical risk or an independent predictor of an adverse clinical outcome. We could not confirm the previously found independent association between the aHb and mortality [5]. The different aHb level groups were not significantly associated with death in the entire group and women (*p*=0.41 and *p*=0.73, respectively), and were only associated with death in men (*p*=0.04) (Table 2). From a clinical point of view, it is irrelevant whether aHb levels are independently associated with a worse outcome or are just a marker for more or less vulnerable patients. Other outcome predictors found in our analysis (Table 2) have firmly established their impact on the outcome [7].

Although this study adds to our knowledge base, there is much that we do not yet understand. It should be noted that our analysis only showed the association between the aHb level and greater MI relative to total cardiac mass and did not establish a causal relationship. This plausible hypothesis may help to understand the possible pathomechanisms for the less favorable outcomes in patients with lower aHb levels, although it is possible that low aHb is not causally related to cardiac injury and that aHb may even be completely independent of outcome (e.g., because of concomitant disease, which is not considered in this analysis). The interplay between aHb levels and comparatively greater MI relative to total cardiac mass, the possible causality, and the underlying mechanisms that are likely multifactorial and contribute to worse outcomes, need further elucidation. The present results should be considered as hypothesis-generating for future research in a similar setting.

### Limitations

There are several limitations to our study. First, this was a single-center, retrospective investigation that allows the generation of a hypothesis, but not the identification of causality. Although the association between aHb and logTn/BSA was assessed with a multivariable model, other potential significant confounders may exist that were not accounted for. Second, we did not measure the heart size and evaluate the left ventricular mass. However, a correlation between BSA and heart weight has been previously well established [13-16]. The majority of peak Tn values were obtained after PCI. The procedure itself could be the reason for higher peak Tn values in patients. We reviewed the possible differences between the aHb groups in relation to multivessel PCI. Numerically, the group with the lowest aHb level had the lowest rate of multivessel PCI, but the difference was not significant (*p*=0.26). In addition, TIMI flow grade just before the start of the PCI procedure showed a J-shaped relationship between aHb and TIMI flow grade (*p*<0.0001), and a similar relationship was observed for TIMI flow grade after PCI (*p*=0.007). In addition, the incidence of anterior wall infarction was similar in all aHb groups (*p*=0.27). These data mean that PCI itself is unlikely to have a significant effect on peak Tn levels in the different aHb groups. Only all-cause mortality was followed, although other potential illnesses associated with lower aHb levels may have influenced the outcome. We lack the data on malignancies, bone marrow diseases, liver dysfunction or liver diseases, and chronic obstructive pulmonary disease. Data are also lacking on smoking, previous MI, heart failure, revascularization, and socioeconomic status, which are known to be powerful predictors of mortality. We did not collect data on medication (except P2Y12 receptor inhibitors during hospitalization), a known factor affecting mortality. Inhibitors of the renin-angiotensin system have a mild hematocrit-lowering effect, which may have confounded our result. In addition, we lack data on iron values. Fifth, the generalizability of our results is questionable because only Caucasians were included. However, there were no exclusion criteria, so this population represents a real experience of patients with MI who require PCI.

## CONCLUSION

Our result suggests that patients with lower aHb levels have lower BSA and lower cardiac mass. A similar Tn peak value in patients with lower aHb levels means relatively greater myocardial injury because of lower cardiac mass. This hypothesis may help to explain the worse outcome in patients with lower aHb levels. According to our result, Tn should be indexed to BSA to provide comparable information on cardiac injury relative to cardiac mass.
